# Pleural Effusion Revealing Visceral Leishmaniasis: A Diagnostic Pitfall in an Immunocompetent Patient

**DOI:** 10.7759/cureus.111717

**Published:** 2026-06-29

**Authors:** Mohammed Aharmim, Siham Mesmoudi, Mohamed Lakhal, Nezha Reguig, Kaoutar Znati, Jamal Eddine El Bourkadi

**Affiliations:** 1 Pulmonology and Phthisiology, Moulay Youssef Hospital, Rabat, MAR; 2 Pathology, Ibn Sina University Hospital, Mohammed V University, Rabat, MAR; 3 Respiratory Diseases, Faculty of Medicine and Pharmacy, Centre Hospitalier Universitaire (CHU) Mohammed VI, Mohammed I University, Oujda, MAR; 4 Pulmonology, Faculty of Medicine and Pharmacy, Moulay Youssef Hospital, Mohammed V University, Rabat, MAR; 5 Pulmonology, Moulay Youssef Hospital, Centre Hospitalier Universitaire (CHU) Ibn Sina, Rabat, MAR

**Keywords:** immunocompetent patient, liposomal amphotericin b, pleural effusion, pleural involvement, visceral leishmaniasis

## Abstract

Pleural involvement in visceral leishmaniasis (VL) is rare and may pose a diagnostic challenge, particularly when it mimics pleural tuberculosis. We report the case of a 39-year-old immunocompetent man who presented with a dry cough and night sweats. Initial workup revealed a small lymphocytic exudative pleural effusion, and pleural biopsy showed granulomatous inflammation with a tuberculoid appearance, leading to empirical antituberculosis treatment. Because of clinical deterioration, persistent pleural effusion, and negative molecular testing for *Mycobacterium tuberculosis*, further investigations were performed. Thoracoabdominal computed tomography demonstrated a moderately sized loculated right pleural effusion without hepatosplenomegaly. Thoracoscopic pleural biopsy revealed intracellular structures compatible with leishmaniasis, and review of the previous specimen confirmed intracytoplasmic Leishman-Donovan bodies within histiocytes. Serology for Leishmania was strongly positive, and treatment with liposomal amphotericin B led to a favorable clinical evolution, although residual pleural thickening persisted. This case underscores the need to consider VL in the differential diagnosis of persistent lymphocytic exudative pleural effusion, even in immunocompetent patients.

## Introduction

Visceral leishmaniasis (VL) is a systemic vector-borne parasitic disease caused by protozoa of the genus Leishmania and transmitted through the bite of infected female phlebotomine sandflies. Despite ongoing control efforts, VL remains a major public health problem in tropical and subtropical regions, including the Mediterranean basin, where *Leishmania infantum* is the predominant species [1].

Classically, VL affects the reticuloendothelial system and presents with prolonged fever, weight loss, hepatosplenomegaly, and hematological abnormalities such as anemia, leukopenia, and thrombocytopenia [2]. However, atypical manifestations may occur and can pose significant diagnostic challenges, particularly when classical systemic features are absent.

Thoracic involvement is uncommon, and pleural manifestations are exceptionally rare. Most reported cases of pleural leishmaniasis have occurred in immunocompromised patients, especially those with HIV infection, whereas only a limited number of cases have been described in immunocompetent individuals [3,4]. In such cases, pleural involvement may represent the predominant or isolated manifestation of the disease, leading to diagnostic confusion with more common conditions, particularly pleural tuberculosis and malignancy.

The distinction between pleural leishmaniasis and pleural tuberculosis may be particularly challenging because both conditions can present with a lymphocytic exudative pleural effusion and granulomatous inflammation. In regions where tuberculosis is highly prevalent, these overlapping features may result in delayed diagnosis and inappropriate treatment. Therefore, microbiological, serological, and histopathological investigations are essential for establishing the correct diagnosis.

We report a rare case of VL with pleural involvement in an immunocompetent patient initially treated for pleural tuberculosis. This case highlights the diagnostic pitfalls associated with this unusual presentation and emphasizes the importance of careful histopathological evaluation and consideration of VL in the differential diagnosis of persistent lymphocytic exudative pleural effusion in endemic areas.

## Case presentation

A 39-year-old man with no significant medical history presented in October 2024 with a dry cough and night sweats that had developed approximately two weeks after prolonged exposure to river sand in an environment favorable to sandfly proliferation. The patient lived in an endemic area for leishmaniasis, making this exposure epidemiologically relevant. Physical examination was otherwise unremarkable, and no peripheral lymphadenopathy or hepatosplenomegaly was detected.

Initial chest radiography revealed a small right pleural effusion (Figure 1). Pleural fluid analysis showed a cloudy citrine-yellow exudate with elevated protein concentration (49 g/L; reference range: <30 g/L for transudative pleural fluid) and lymphocytic predominance (90%). Histopathological examination of a pleural biopsy demonstrated granulomatous inflammation with a tuberculoid appearance, raising suspicion of pleural tuberculosis and prompting initiation of standard first-line anti-tuberculosis therapy.

**Figure 1:**
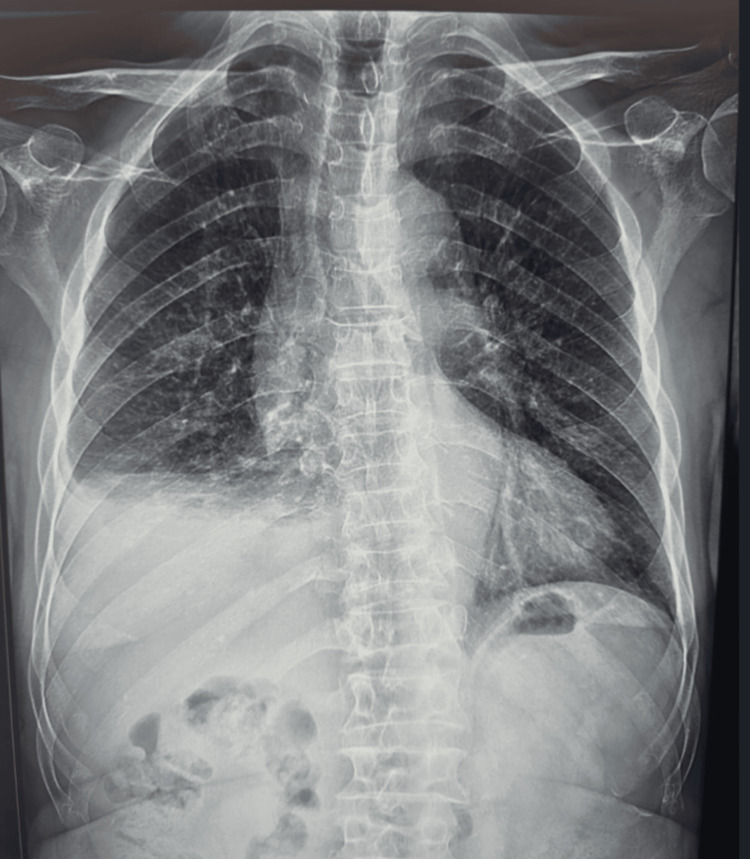
Initial chest radiograph demonstrating a small right pleural effusion.

Over the following month, the patient's condition progressively worsened, with exertional dyspnea, asthenia, and a 3-kg weight loss, accompanied by an increase in pleural effusion volume. In the absence of clinical improvement, anti-tuberculosis treatment was discontinued after six weeks.

The pleural effusion persisted and required repeated drainage procedures. Repeat pleural fluid analysis confirmed a lymphocyte-predominant exudate (protein: 42 g/L; reference range: <30 g/L for transudative pleural fluid; lactate dehydrogenase: 692 U/L; reference range: 135-225 U/L; lymphocytes: 98%), with sterile cultures and elevated adenosine deaminase (ADA) levels (81 U/L; reference range: <40 U/L). Polymerase chain reaction testing for *Mycobacterium tuberculosis* in both sputum and pleural fluid (Xpert MTB/RIF; Cepheid, Sunnyvale, CA, USA) was negative. A second pleural biopsy revealed only non-specific inflammatory changes.

Thoracoabdominal computed tomography demonstrated a moderately sized loculated right pleural effusion without hepatosplenomegaly (Figure 2). Owing to the persistence of the effusion and the lack of response to empirical anti-tuberculosis treatment, a surgical pleural biopsy was performed by video-assisted thoracoscopy. Histological examination revealed epithelioid and giant-cell granulomatous lesions containing numerous intracellular rounded structures suggestive of leishmaniasis. Retrospective review of the previous biopsy specimens subsequently identified intracytoplasmic Leishman-Donovan bodies within histiocytes, confirming pleural involvement by VL.

**Figure 2:**
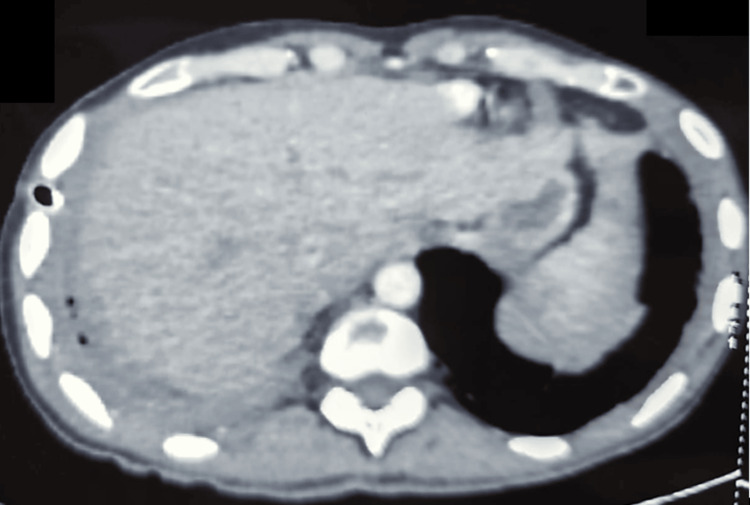
Thoracoabdominal CT showing a moderately sized loculated right pleural effusion without hepatosplenomegaly.

Leishmania serology was strongly positive (IgG + IgM = 10.59; cutoff value = 1.1). Laboratory investigations showed moderate lymphopenia (0.9 × 10⁹/L; reference range: 1.0-4.0 × 10⁹/L), mild elevation of inflammatory markers (C-reactive protein: 10.8 mg/L; reference range: <5 mg/L), and polyclonal hypergammaglobulinemia (18 g/L; reference range: 7-16 g/L). Serological tests for HIV, hepatitis B virus, and hepatitis C virus were negative.

Based on the histopathological identification of Leishman-Donovan bodies and strongly positive serology, a diagnosis of VL with pleural involvement was established approximately three months after the initial presentation. Bone marrow examination was not performed because the diagnosis had already been confirmed, and no severe hematological abnormalities were present.

The patient received liposomal amphotericin B (AmBisome®) at a cumulative dose of 18-24 mg/kg administered over six infusions according to current recommendations. Clinical evolution was favorable, with progressive resolution of symptoms. Follow-up chest radiography demonstrated residual right pleural thickening (Figure 3).

**Figure 3:**
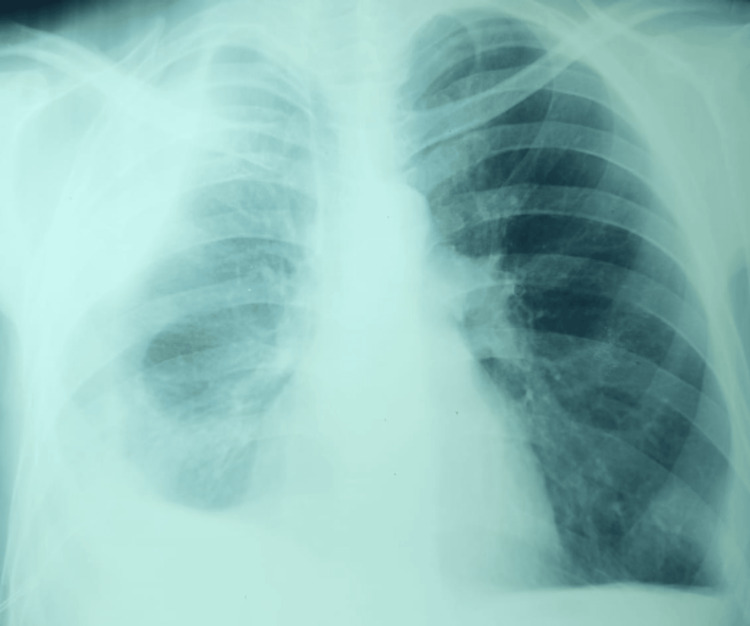
Follow-up chest radiograph showing residual right pleural thickening.

## Discussion

VL is a potentially life-threatening parasitic disease caused predominantly by *Leishmania infantum* in Mediterranean countries [1,2]. Despite ongoing control efforts, VL remains endemic in several regions worldwide and continues to represent a significant public health concern, particularly in the Mediterranean basin [3]. Classical manifestations include prolonged fever, constitutional symptoms, hepatosplenomegaly, pancytopenia, and polyclonal hypergammaglobulinemia [1,2]. However, atypical presentations lacking these characteristic features have been increasingly recognized. In our patient, neither hepatosplenomegaly nor pancytopenia was present, illustrating that their absence does not exclude the diagnosis when compatible epidemiological and histopathological findings are present.

Thoracic involvement is uncommon, and pleural manifestations are exceptionally rare [2,4-6]. Most published cases have been reported in immunocompromised patients, particularly those with HIV infection [5-7]. Chenoweth et al. described AIDS-related VL presenting with bilateral pleural effusion, with direct identification of amastigotes in pleural fluid [6]. Likewise, Diehl et al. demonstrated *Leishmania chagasi* in pleural and ascitic fluids using microscopy and polymerase chain reaction [5]. Among immunocompetent individuals, only isolated case reports are available. Dasgupta et al. described an immunocompetent patient with isolated pleural effusion [4]. Similar to that observation, our case demonstrates that pleural involvement may represent the predominant manifestation of VL, even in the absence of overt systemic disease, thereby expanding the spectrum of atypical presentations.

The exact mechanisms responsible for pleural localization remain incompletely understood. Proposed explanations include hematogenous dissemination of infected macrophages, extension from subclinical pulmonary involvement, and host-mediated inflammatory responses [2,7]. However, these mechanisms remain largely hypothetical and should be interpreted cautiously because direct evidence supporting these pathways remains limited.

One of the major diagnostic challenges in the present case was the close resemblance to pleural tuberculosis. Lymphocytic exudative pleural effusion, granulomatous inflammation, and markedly elevated ADA levels initially favored the diagnosis of tuberculosis, leading to empirical anti-tuberculosis treatment. Similar diagnostic difficulties were reported by Dasgupta et al. [4]. Nevertheless, elevated ADA levels should not be considered diagnostic of tuberculosis in isolation. Although ADA values above 40 U/L strongly support tuberculous pleurisy, increased levels may also occur in several non-tuberculous conditions, including empyema, lymphoma, connective tissue diseases, and certain infectious or inflammatory disorders [8,9]. Therefore, ADA should always be interpreted in conjunction with clinical, microbiological, and histopathological findings rather than as a standalone diagnostic marker [8,9]. Indeed, despite the high diagnostic performance of pleural fluid ADA, a definitive diagnosis still relies on microbiological or histopathological confirmation because false-positive results may occur [8,9].

Repeated negative Xpert MTB/RIF assays and the absence of clinical improvement after six weeks of anti-tuberculosis therapy prompted further investigations. Histopathological examination of the surgical pleural biopsy demonstrated numerous Leishman-Donovan bodies within histiocytes, and strongly positive serology further supported the diagnosis. A retrospective review of the initial biopsy specimens subsequently revealed previously overlooked amastigotes. The organisms were probably missed initially because granulomatous inflammation with a tuberculoid appearance dominated the histological picture, and the rarity of pleural leishmaniasis reduced diagnostic suspicion. This observation emphasizes the importance of careful pathological assessment and expert slide review when evaluating atypical granulomatous lesions.

Definitive diagnosis of VL relies on direct demonstration of the parasite and may be supported by serological and molecular techniques [10,11]. Available diagnostic methods include microscopy, culture, serology, and polymerase chain reaction [5,10]. In the case reported by Diehl et al., Leishmania DNA was successfully detected in pleural and ascitic fluids, illustrating the usefulness of molecular techniques in unusual presentations [5]. Nevertheless, histopathological examination remains a cornerstone of diagnosis when tissue samples are available. In our patient, the identification of Leishman-Donovan bodies within pleural histiocytes provided definitive evidence of pleural involvement. Although bone marrow examination is traditionally considered a reference diagnostic procedure, it was not performed because histopathological examination of pleural tissue had already established the diagnosis, and severe hematological abnormalities were absent [10,12].

Current guidelines recommend liposomal amphotericin B as the treatment of choice because of its high efficacy and favorable safety profile [11-13]. Alternative agents include pentavalent antimonials, miltefosine, and paromomycin, although toxicity concerns and emerging resistance have limited their use [13,14]. In our patient, treatment with liposomal amphotericin B resulted in favorable clinical evolution.

Published outcomes are heterogeneous, ranging from complete recovery to recurrent pleural disease in immunocompromised patients [4-7,15]. Residual pleural thickening persisted in our patient, suggesting that delayed diagnosis and prolonged inflammation may contribute to irreversible pleural remodeling. Such sequelae have only rarely been emphasized in previously reported cases.

Several diagnostic pitfalls contributed to the delayed diagnosis in the present case, including the rarity of pleural involvement, the absence of classical manifestations such as hepatosplenomegaly and pancytopenia, elevated ADA levels, granulomatous inflammation suggestive of tuberculosis, and the initial failure to recognize amastigotes on biopsy specimens. These factors highlight the importance of maintaining a broad differential diagnosis in endemic regions.

This case provides additional evidence that VL should be considered among the differential diagnoses of persistent lymphocytic exudative pleural effusion, particularly in endemic areas and in patients who fail to respond to empirical anti-tuberculosis therapy. Early recognition, careful histopathological evaluation, and appropriate ancillary testing are essential to avoid diagnostic delay and improve outcomes.

## Conclusions

Pleural VL is a rare and underrecognized presentation that may result in diagnostic delay and inappropriate treatment. This case emphasizes that VL should be considered in the differential diagnosis of atypical lymphocytic exudative pleural effusion, particularly when there is no response to empirical anti-tuberculosis therapy, even in immunocompetent patients.

The diagnosis relies on direct demonstration of the parasite, with histopathological examination remaining the cornerstone of confirmation and molecular methods serving as useful adjuncts in selected cases. Early recognition is essential to ensure timely initiation of appropriate treatment, most commonly liposomal amphotericin B, and to potentially limit pleural sequelae. Improved awareness of this unusual presentation may help clinicians avoid misdiagnosis and optimize patient care.
